# Cardiovascular disease prevalence in patients with inflammatory arthritis, diabetes mellitus and osteoarthritis: a cross-sectional study in primary care

**DOI:** 10.1186/1471-2474-13-150

**Published:** 2012-08-21

**Authors:** Markus MJ Nielen, Alper M van Sijl, Mike JL Peters, Robert A Verheij, François G Schellevis, Michael T Nurmohamed

**Affiliations:** 1 NIVEL (Netherlands Institute for Health Services Research), P.O. Box 1568, 3500BN, Utrecht, The Netherlands; 2 Department of Rheumatology, Reade, Amsterdam, The Netherlands; 3 Department of Internal Medicine, VU University Medical Center, Amsterdam, The Netherlands; 4 Department of General Practice / EMGO Institute, VU University Medical Center, Amsterdam, The Netherlands

## Abstract

**Background:**

There is accumulating evidence for an increased cardiovascular burden in inflammatory arthritis, but the true magnitude of this cardiovascular burden is still debated. We sought to determine the prevalence rate of non-fatal cardiovascular disease (CVD) in inflammatory arthritis, diabetes mellitus and osteoarthritis (non-systemic inflammatory comparator) compared to controls, in primary care.

**Methods:**

Data on CVD morbidity (ICPC codes K75 (myocardial infarction), K89 (transient ischemic attack), and/or K90 (stroke/cerebrovascular accident)) from patients with inflammatory arthritis (n = 1,518), diabetes mellitus (n = 11,959), osteoarthritis (n = 4,040) and controls (n = 158,439) were used from the Netherlands Information Network of General Practice (LINH), a large nationally representative primary care based cohort. Data were analyzed using multi-level logistic regression analyses and corrected for age, gender, hypercholesterolemia and hypertension.

**Results:**

CVD prevalence rates were significantly higher in inflammatory arthritis, diabetes mellitus and osteoarthritis compared with controls. These results attenuated - especially in diabetes mellitus - but remained statistically significant after adjustment for age, gender, hypertension and hypercholesterolemia for inflammatory arthritis (OR = 1.5 (1.2-1.9)) and diabetes mellitus (OR = 1.3 (1.2-1.4)). The association between osteoarthritis and CVD reversed after adjustment (OR = 0.8 (0.7-1.0)).

**Conclusions:**

These results confirm an increased prevalence rate of CVD in inflammatory arthritis to levels resembling diabetes mellitus. By contrast, lack of excess CVD in osteoarthritis further suggests that the systemic inflammatory load is critical to the CVD burden in inflammatory arthritis.

## Background

In inflammatory arthritis patients the cardiovascular disease (CVD) burden is more prevalent, best exemplified by higher rates of cardiovascular mortality and morbidity in rheumatoid arthritis (RA) patients [1-3]. The increased CVD risk in inflammatory arthritis patients can not be fully explained by classical risk factors [4,5]. Additionally, chronic inflammation either directly or indirectly via insulin resistance, endothelial dysfunction, and dyslipidaemia, has emerged as potential mechanism for the excessive CVD risk in inflammatory arthritis patients [6].

The true magnitude of the cardiovascular burden in inflammatory arthritis is still debated, since outcomes of studies assessing CVD prevalence and incidence are difficult to compare due to differences in study design and study populations. Most studies are performed in secondary care-based cohorts and only a minority in community-based settings, which, as a consequence, may lead to an overestimation of the CVD burden due to an overrepresentation of inflammatory arthritis patients with more severe disease [1,7,8]. This problem can be solved by using data from electronic medical records (EMRs) from general practitioners (GPs). Inflammatory arthritis patients in primary care have a broader spectrum of disease severity, which may result in a more accurate estimate of the CVD prevalence. In the Netherlands, GPs have a gatekeeper role for access to specialized care. All Dutch inhabitants are listed with a general practice, and the GP is the first professional to be consulted for health problems.

Using data from GPs also makes it possible to compare morbidity and health care utilization of inflammatory arthritis patients with other patient groups or healthy individuals. This latter possibility is interesting, because we recently observed that the magnitude of CVD risk in rheumatoid arthritis patients approached that of diabetes mellitus [9,10], a well-established cardiovascular risk factor. In these studies RA patients in specialized care were compared with diabetes mellitus patients from a population-based cohort. Using data from patients in primary care may result in a better estimation of the CVD risk of inflammatory arthritis and diabetes mellitus patients, because these patient populations are representative for the total patient population.

Additionally, it would not only be interesting to compare CVD risk between inflammatory arthritis and diabetes mellitus patients, but also to include a group of osteoarthritis patients and controls. Using controls makes it possible to compare the CVD risk in the patient groups with ´healthy individuals. If indeed inflammation enhances CVD risk, one may hypothesize that osteoarthritis of the knee and/or hip (a non-systemic ‘inflammatory’ arthritis) will be associated with a less pronounced CVD burden. Against this background, we determined the non-fatal CVD prevalence rate in patients with inflammatory arthritis, diabetes mellitus, and osteoarthritis, as compared to patients without these disorders, in primary care.

## Methods

### Study population

Data were used from the Netherlands Information Network of General Practice (LINH). These data were retrieved from EMRs kept by a nationally representative sample of approximately 160 GPs working in 96 general practices with 360,000 registered patients in 2006. Data include information on consultations, morbidity, prescriptions and referrals to other healthcare professionals. Practices as well as patients are representative for the Dutch population [11,12].

Diagnoses were recorded using the International Classification of Primary Care (ICPC-1) coding system [13]. When issuing a prescription, a diagnostic code was recorded, and the selected drug was automatically linked to the Anatomical Therapeutic Chemical (ATC) coding system (http://www.whocc.no). In this study morbidity data and data on all prescriptions issued by the participating practices were used. Only individuals of 30 years and older were included in the statistical analyses, because individuals below the age of 30 years have a lower probability of having CVD [14], but also inflammatory arthritis, osteoarthritis, and/or diabetes mellitus. Practices that recorded data during less than six months in 2006 were also excluded from the analyses, because the selection of the patient groups are based on complaints and diseases presented during GP consultations. When more than half of the consultations in a year are missing, the prevalence rates of the studied diseases are underestimated.

### Population selection

Based on the ICPC coded morbidity and prescription data, it was determined whether the participating patients had any of the following diagnoses in 2006: 1) inflammatory arthritis (ICPC code L88; rheumatoid arthritis or ankylosing spondylitis), 2) diabetes mellitus (ICPC code T90), 3) osteoarthritis of knee and/or hip (ICPC code L89 and/or L90), 4) CVD (ICPC codes K75 (myocardial infarction), K89 (transient ischemic attack), and/or K90 (stroke / cerebrovascular accident), 5) hypertension (ICPC code K86 and/or K87) and 6) hypercholesterolemia (ICPC code T93). Individuals were classified within the above mentioned groups, when the diagnosis was recorded in 2006 or in previous years (up to 2004). By using a period of three years, we could determine the diagnoses more reliable, since not all patients are visiting their GP every year with complains related to the studied diseases.

It was not possible to discriminate between rheumatoid arthritis and ankylosing spondylitis within the group of inflammatory arthritis patients and between diabetes mellitus type 1 and 2. Per patient we also determined whether they used cardio protective medication (statins and/or anti-hypertensive agents) in 2006. All other patients listed in the participating practices without inflammatory arthritis, diabetes mellitus and/or osteoarthritis were used as controls.

### Statistical analyses

First, CVD prevalence rates were calculated among individuals with inflammatory arthritis, diabetes mellitus, osteoarthritis and controls. Differences in age, gender, the prevalence rates of CVD, hypertension and hypercholesteremia and the use of statins and antihypertensives between the three patient groups and controls were tested with Student’s t-test and chi-square tests.

Second, the association between the presence of CVD and the presence of the three diseases was determined with multilevel logistic regression analyses with a random intercept using the second order PQL method [15] due to the hierarchical structure of the data (patients clustered in practices). Three models were calculated: 1) a model with the three studied independent variables (presence of inflammatory arthritis, diabetes mellitus and osteoarthritis), 2) model 1 plus age and gender, and 3) model 2 plus CVD risk factors (hypertension and hypercholesterolemia). Results are presented as odds ratios with 95%-confidence intervals. Interaction terms were introduced to test for selective amplification of the CVD risk by subjects with more than one of the investigated diseases and the possible modifying effect of age.

Multilevel logistic regression analyses were performed with MLwiN, a statistical program for multilevel analyses [16]. Student’s t-test and chi-square tests were performed with Stata10.

## Results

### Population characteristics

Data were used from 175,061 patients in 69 general practices. Of these patients, 1,518 patients (0.9%) were diagnosed with inflammatory arthritis, 11,959 (6.8%) with diabetes mellitus, and 4,040 (2.3%) with osteoarthritis. The remaining 158,439 individuals were used as controls. Because patients can have multiple disorders, the sum of the number of patients in the four groups exceeds the total of 175,061 patients. The characteristics of the patient groups and controls are shown in Table 1.

**Table 1:** Population characteristics

	**IA**	**DM**	**OA**	**Controls**
	**(n = 1,518)**	**(n = 11,959)**	**(n = 4,040)**	**(n = 158,439)**
Mean age, years	60.3**	65.8**	69.8**	51.0
Female gender,%	64.1**	51.9*	68.7**	50.4
AMI,%	1.8**	2.8**	1.5**	0.7
TIA,%	2.0**	2.2**	2.0**	0.6
CVA,%	3.0**	3.7**	2.7**	0.9
Total CVD,%	6.5**	8.1**	5.8**	2.0
Hypertension,%	30.9**	47.7**	38.5**	13.3
Hypercholesterolemia,%	10.8**	24.0**	13.3**	4.8
Use of anti-hypertensive agents,%	36.4**	60.6**	45.0**	14.6
Use of statins,%	15.7**	48.8**	20.0**	6.0
Inflammatory arthritis,%	—	1.5	1.6	—
Diabetes mellitus,%	11.5	—	16.5	—
Osteoarthritis,%	4.4	5.6	—	—

* p < 0.01; ** p < 0.001 (compared to controls) DM, diabetes mellitus; IA, inflammatory arthritis; OA, knee- and/or hip osteoarthritis; AMI, acute myocardial infarction; TIA, transient ischemic attack; CVA, cerebrovascular accident; CVD, cardiovascular disease.

Compared with controls, individuals with inflammatory arthritis, diabetes mellitus and osteoarthritis were older and had higher prevalence rates of CVD, hypertension and hypercholesterolemia. Also, individuals with inflammatory arthritis, diabetes mellitus and osteoarthritis were more often female and used significantly more statins and anti-hypertensive agents compared with the control group. The prevalence rate of diabetes mellitus and osteoarthritis in inflammatory arthritis was 11.5% and 4.4%, respectively.

### Association between inflammatory arthritis, diabetes, osteoarthritis and CVD

Significantly higher CVD rates were found in individuals with inflammatory arthritis (OR = 2.1), diabetes mellitus (OR = 3.7), and osteoarthritis (OR = 1.9) compared with controls (model 1, Table 2). After adjustment for age, gender and CVD risk factors (hypertension and hypercholesterolemia; model 3), both inflammatory arthritis and diabetes mellitus were still positively associated with CVD, but to a lesser extent. We found a larger association between inflammatory arthritis and CVD (OR = 1.5) than between diabetes mellitus and CVD (OR = 1.3). The association between osteoarthritis and CVD reversed after adjustment for age, gender and CVD risk factors (OR = 0.8). Interaction terms were added to model 3 to test for additional effects for subjects with more than one of the investigated diseases and the modifying effect of age. However, none of the interaction terms reached statistical significance (data not shown).

**Table 2:** Association between CVD and inflammatory arthritis, diabetes and osteoarthritis

	**Controls**	**IA**	**DM**	**OA**
Model 1	1.0	2.1 (1.7-2.7)*	3.7 (3.5-4.0)*	1.9 (1.6-2.2)*
Model 2	1.0	1.6 (1.3-2.0)*	1.8 (1.6-1.9)*	0.9 (0.8-1.0)
Model 3	1.0	1.5 (1.2-1.9)*	1.3 (1.2-1.4)*	0.8 (0.7-1.0)*

* p <0.001 Results as odds ratios (95%-confidence interval) DM, diabetes mellitus; IA, inflammatory arthritis; OA, knee and/or hip osteoarthritis.

Model 1: Unadjusted for risk factors.

Model 2: Model 1 adjusted for age and gender.

Model 3: Model 1 adjusted for age, gender, hypertension and hypercholesterolemia.

Also, subgroup analyses were performed with myocardial infarction (MI), transient ischemic attack (TIA), and cerebrovascular accident (CVA) as dependent variables. After correction for confounders, the association of OA with these CVD subgroups did not differ from the main effects, although due to less power these effects did not reach statistical significance. MI, TIA and CVA were all positively associated with IA (OR = 1.3, OR = 1.4 and OR = 1.5, respectively), although only the association between IA and CVA was statistically significant. For diabetes mellitus, we found statistically significant associations with MI and CVA (OR = 1.3 and OR = 1.4, respectively).

## Discussion

There is considerable interest in the association between inflammatory arthritis and cardiovascular disease, but evidence from primary care settings emphasizing this association is sparse. To the best of our knowledge, this is the first study to report on the CVD prevalence rate in inflammatory arthritis, diabetes mellitus and osteoarthritis patients compared with controls in a nationally representative primary care population. We report nearly identical age, sex, hypertension and hypercholesterolemia adjusted CVD prevalence rates in patients with either inflammatory arthritis or diabetes mellitus, supporting our previous observations that the magnitude of CVD burden in inflammatory arthritis is comparable with that in diabetes mellitus [9,10].

The fact that adjustment for hypertension and hypercholesterolemia attenuated the association between CVD and diabetes mellitus, but far less so the association between CVD and inflammatory arthritis, is another important observation. Although it is not possible to report cause-and-effect relationships with the cross-sectional design, there are two possible explanations for this observation. First, given the lower prevalence rate of hypertension and hypercholesterolemia and lower use of statins and anti-hypertensive agents in inflammatory arthritis patients compared with diabetes mellitus, one may speculate that classic risk factors are underreported and hence undertreated in inflammatory arthritis. CVD risk management is one of the main goals in the treatment of diabetes mellitus, but, at least until recently, not in inflammatory arthritis patients, which supports this hypothesis. Second, factors other than classic CVD risk factors potentially explain the strong association between inflammatory arthritis and CVD. Indeed, it is increasingly hypothesized that individuals with inflammatory arthritis are more affected by CVD because of a chronic systemic inflammatory process, as this accelerates the development of all stages of atherosclerosis [17]. Inflammatory cells are commonly found within atherosclerotic lesions, particularly in the culprit lesions, and many inflammatory cells are implicated in the pathogenesis of atherosclerosis from its earliest stages [6]. Additionally, inflammation may indirectly increase CVD risk through deteriorating CVD risk factors [18]. The fact that osteoarthritis (a non-systemic inflammatory comparator) did not convey any excess CVD risk after adjustment for CVD risk factors is another important observation and may comply with the inflammatory hypothesis. Surprisingly, after adjustment, osteoarthritis was negatively associated with CVD. A possible explanation for this finding is selective drop-out (‘healthy survivors effect’) in the group osteoarthritis patients. Because of the high mean age of this group (about 70 years old) it is likely that the patients with the highest risks already died, resulting in an underrepresentation of the prevalence rate of CVD.

Using data from electronic medical records from general practices makes it possible to compare the prevalence of CVD between different patient groups in the same study population. This has the advantage that all morbidity is measured in the same manner, minimizing selection bias. Another strength of this study is the use of a representative group of patients with a mean average disease severity. The fact that the observed CVD prevalence rates in inflammatory arthritis and diabetes mellitus concur with other studies lends external validity to our observations [7]. Also, of important note is the adjustment for important CVD risk factors (age, gender, hypertension, and hypercholesterolemia) on the one hand, and controlling for amplification of CVD risk by individuals with more than one disease on the other hand. Some other studies also reported an increased prevalence of CVD in inflammatory arthritis from a primary care population, but neither of these studies controlled for hypertension and/or hypercholesterolemia [7,8].

Besides the advantage of having access to large representative patient populations, using data from health registries also have some disadvantages. First, the large number of patients makes it impossible to validate diagnoses and to collect additional data in terms of time and funding. Although diagnoses are not validated by a medical specialist, the used GP diagnoses are based on the guidelines of the Dutch College of General Practitioners (NHG). Furthermore, medical specialists send a letter to the GP after the consultation of a patient with information about diagnosis and treatment. It is likely that the GP uses this information to register the diagnosis inflammatory arthritis in the EMR of the patient. Therefore, we expect an overestimation of the number of IA patients in our primary care cohort, which might resulted in an underestimation of our results. Second, data in health registries are not collected in a structural way and patients are not measured periodically. Data are only available from patients who visit their GP for a health problem. As a results, important confounders, such as smoking and obesity, are not always registered and these variables cannot be used in the statistical analyses. Also, not all prescribed drugs in specialised care are registered in the EMRs of GPs, which makes it not possible to measure the use of DMARDs reliably. This hampers us to adjust for these potentially important confounders (residual confounding). A limitation of the cross-sectional design is a lack of information on the sequence of the disease and CVD events, and hence a causal relationship between the advent of inflammatory arthritis and development of CV disease can not be proven.

## Conclusions

In conclusion, this is the first study demonstrating an increased prevalence rate of CVD in inflammatory arthritis to levels broadly similar to diabetes mellitus in a representative primary care population. The lack of excess CVD in osteoarthritis further suggests that systemic inflammatory load appears critical to the CVD burden in inflammatory arthritis and indirectly underscores the importance of adequate control of disease activity in inflammatory arthritis patients to lower CVD risk.

## Competing interests

The authors declare that they have no competing interests.

## Authors' contributions

MMJN performed the study according to protocol, analyzed the data and wrote the manuscript. AMvS and MJLP helped writing the manuscript and contributed substantially to the statistical analysis and interpretation of the data. FGS, RAV, MTN supervised the study and developed the study protocol. All authors read and approved the final manuscript.

## Pre-publication history

The pre-publication history for this paper can be accessed here:

http://www.biomedcentral.com/1471-2474/13/150/prepub
